# Immigration, citizenship, and the mental health of adolescents

**DOI:** 10.1371/journal.pone.0196859

**Published:** 2018-05-03

**Authors:** Nicole Filion, Andrew Fenelon, Michel Boudreaux

**Affiliations:** Department of Health Services Administration, University of Maryland School of Public Health, College Park, Maryland, United States of America; Universidad del Desarrollo, CHILE

## Abstract

**Purpose:**

To examine the reported mental health outcomes of adolescent foreign-born non-citizens and adolescent foreign-born U.S. citizens compared to adolescent U.S.-born citizens.

**Methods:**

Using the Strengths and Difficulties Questionnaire in the National Health Interview Survey, we compared mental health status of U.S.-born adolescent citizens to foreign-born citizens and non-citizens in the years 2010–2015, and examined how differences in emotional difficulty changed based on time spent in the U.S.

**Results:**

Results suggest that non-citizen adolescents experience better mental health outcomes than U.S.-born citizens. However, the mental health status of foreign-born citizens is indistinguishable from that of the U.S.-born, after accounting for basic socio-demographic characteristics. The prevalence of emotional difficulty experienced by immigrant adolescents increased with a family’s duration in the U.S.

**Conclusion:**

Our findings are consistent with a broader health advantage for the foreign-born, but we present new evidence that the mental health advantage of foreign-born adolescents exists only for non-citizens.

## Introduction

Adolescence is a time of “storm and stress” in child development.[1] New-found independence, the increased importance of peer interaction, and substantial physical and mental development increases the risk of depression, anxiety and other mental health problems.[2] Indeed, adolescence is the most common time in an individual’s life for psychiatric illness to emerge.[3] Not only is mental health status more likely to emerge during adolescence, but under-treated mental health problems during adolescence increase the risk of negative outcomes throughout the life-course, including disability, loss of future productivity and contribution to the community, lower educational achievement, and a higher likelihood of risky behaviors.[4]

Immigrant adolescents would seem to be at particularly elevated risk of experiencing mental health problems given the unique and precarious position of the foreign-born in American society. However, much of the previous literature on immigrant mental health has considered all foreign-born adolescents as a singular group, and surprisingly little is known about the role of citizenship status in impacting the mental health status of immigrant adolescents. In the U.S. today, the immigrant population is comprised of naturalized U.S. citizens (44.1%), lawful permanent residents (26.6%), unauthorized immigrants (24.5%), and temporary lawful residents (4.8%).[5]

While citizenship is often overlooked, existing literature that examines the mental health status of immigrant children and adolescents tends to find a foreign-born advantage. A 2013 study examined nativity differences in children and found that immigrant children experience a lower prevalence of depression and behavioral problems.[6] One study in Southern Florida found that foreign-born Latino high school students have a lower prevalence of substance use disorders than U.S.-born Latino high school students.[7] Another study that uses nationally representative data found that in the 1990’s first generation immigrant youth experienced lower levels of depression and higher levels of positive well-being than similar native-born peers, but the advantage did not persist for second generation immigrants.[8]

Other literature in this field has studied the mental health and well-being of youth with undocumented parents. Findings suggest that parents’ unauthorized status is a substantial barrier to healthy child development and perpetuates health inequalities in this population. [9] Studies have looked at the mental health of undocumented children[10], but the role of citizenship in the mental health of children and adolescents has received little attention. This is an important gap in the literature given that over half of the non-citizen populations are lawful immigrants. Several other important questions also remain unanswered, such as how mental health outcomes evolve with longer time spent in the U.S. and if outcomes today differ from those observed in the 1990’s, when both the immigrant population and immigration policy were substantially different.[11]

Immigrants to the United States often arrive with relatively low socioeconomic status and come from countries with poorer population health outcomes than the U.S.[12] Despite these challenges, immigrants tend to exhibit better health and mortality outcomes than the native born, a pattern referred to as the “immigrant paradox.”[13] Adult immigrants, on average, also tend to report better mental health and lower rates of depression and anxiety than U.S.-born.[14] These advantages are thought to be the result of several factors, including selective migration and a high level of community social support. For many health outcomes, this advantage fades with greater time spent in the U.S., and may disappear for the second generation.[15]

While immigrant communities may possess a set of general protective factors, risk factors for mental health problems that are common during adolescence are particularly elevated among the immigrant population. For example, first generation immigrants are more likely than non-immigrant U.S.-citizens and second or third generation immigrants to experience discrimination, peer aggression and socioeconomic disadvantage, factors that have been shown to decrease psychosocial wellbeing. [16] Furthermore, they undergo the stress of language barriers and acculturation to the U.S.[17]

Immigrant youth must contend with highly uncertain and restrictive immigration policies, particularly evident in the recent debate regarding the Deferred Action for Childhood Arrivals (DACA) program, [10] which the President cancelled before quickly issuing a public statement expressing his desire to extend protections for “DREAMers.”[18] With the rising social and policy pressures immigrant adolescents face, it remains unclear how the protective factors underlying the “immigrant paradox” balance against the role of citizenship status in the immigrant population. Citizenship represents an officially sanctioned integration into U.S. society. Citizenship grants fuller access to public benefits and economic opportunities, it may serve as a marker for cultural assimilation, and foreign-born citizens may experience a different level or type of animus than non-citizens. All of these factors may contribute to differences in adolescent mental health.

Race may interact with immigration and citizenship status in two important ways. First, race is highly correlated with an immigrant’s global region of origin, which might be related to mental health. In recent decades as immigration from Asia, Africa, and the Caribbean has grown, the racial and ethnic distribution of foreign-born children and adolescents has become more diverse.[5] Second, race presents an additional dimension of inequality that immigrants must contend with. Immigrant adolescents may face racial discrimination as they integrate into the American stratification system,[19] and it is important consider racial differences in the roles of nativity and citizenship in adolescent mental health.

In this study, we used a nationally representative survey to examine how the reported mental health status of foreign-born non-citizens and foreign-born U.S. citizens compare to those of U.S.-born citizens. We also examined whether duration in the U.S. is associated with the prevalence of mental health problems and if the association of immigration and the outcomes of interest varied across racial groups.

## Data and methods

Data on adolescents (age 10–17) came from a harmonized version of the 2010–2016 National Health Interview Survey (NHIS).[20] The NHIS is an annual cross-sectional household survey that is drawn from the civilian non-institutionalized U.S. population and sponsored by the National Center for Health Statistics. The survey is comprised of a core set of questions that contains basic demographic and health information for each member of the household. A knowledgeable adult provides more detailed information about the health and well-being of one randomly selected child per household (“the sample child”). Overall, the annual household response rate is approximately 80%.

After removing cases with missing values (N = 749), our final analytic sample consisted of 39,918 adolescents, including 36,937 U.S.-born citizens, 1,085 foreign-born U.S. citizens, and 1,896 non-citizens (including lawful and unauthorized immigrants). All analyses were weighted and accounted for in the complex sample design of the NHIS.

### Immigrant status

The primary independent variable measuring place of birth and citizenship status consisted of the following categories: U.S.-born citizen, foreign-born U.S. citizen, and non-citizen. The non-citizen group includes lawful residents, refugees, students, temporary workers, and undocumented immigrants. The foreign-born citizen group includes all foreign-born individuals that are naturalized U.S. citizens.

### Mental health status

The outcome of interest was mental health status, which was measured using three separate variables. The first measure was the summary score from the Strengths and Difficulties Questionnaire (SDQ), which is a mental health scale consisting of 5 items.[21] Scale scores range from 0–10, with higher scores indicating worse mental health. The SDQ is highly predictive of mental illness and mental health service use, is a valid measure with proxy informants, and has been used across diverse populations. [22,23] The use of proxy informants, generally a knowledgeable adult or parent, when reporting on child and adolescent outcomes tends to be approach.[24] The second measure was a 0/1 indicator of likely psychological problems, defined as an SDQ score above 6.[24] Our final outcome was a 0/1 indicator of emotional difficulties from a question that asks whether the child experiences any emotional difficulties. The mental health outcomes we used were based on reported symptoms rather than diagnoses and so are not dependent on health care access.

### Covariates

We adjusted for basic socio-demographic covariates that may be associated with mental health and immigration status. These included age (10–12, 13–15, 16–17), sex, race/ethnicity (Non-Hispanic White, Non-Hispanic Black, Non-Hispanic Asian, Hispanic, Non-Hispanic Other), family income to poverty ratio (<200% FPL, 200–400% FPL, 400+ FPL), and the highest educational attainment in the family (high school or less, some college, college degree or more).

## Analytical approach

We used multivariable regression to examine citizenship/place of birth differences for each outcome. In these models, we adjust for age, race and ethnicity, gender, income, and highest household educational attainment. Continuous SDQ scores were modeled using linear regression and likely psychological problem and emotional difficulty were modeled using logistic regression. After estimating models for the full target population, we repeated the analysis after stratifying the sample by race/ethnicity to determine if associations differed across racial groups.

Next, using a similar regression approach, we examined if a family’s duration in the U.S. was associated with the likelihood of experiencing emotional difficulties (the most prevalent of our outcomes). Family duration was defined as the maximum duration of a member of the household, categorized into three bins (<5 years, 5–10 years, >10 years), and reflects the environment of the family rather than child’s specific migration experience. To account for small sample sizes, this analysis combined the foreign-born citizen and non-citizen categories and kept U.S.-born citizens in their own category. Focusing on the household duration rather than the child’s was necessary to account for the fact the data include only broad measures of time (<5 years, 5–10 years, >10 years) that are mechanically correlated with adolescent age.

Finally, we conducted a set of sensitivity analyses. We examined if results varied after controlling for language of the interview, how sensitive our results are to the inclusion of poverty as a covariate, how sensitive our results were to including Puerto Ricans in the Hispanic category, and if results changed excluding the years after 2013, when the Affordable Care Act may have influenced outcomes differently for citizen and non-citizen youth. The sensitivity analyses are described in more detail in the Supplementary Tables (S2 Table through S5 Table).

## Results

Table 1 displays descriptive statistics of adolescents by immigrant status. There were large differences between groups in the racial and ethnic distribution. The majority (59.0%) of U.S.-born citizens were Non-Hispanic Whites compared to 29.0% for foreign-born citizens and 11.6% for non-citizens. In contrast, Hispanic individuals comprised 20.5% of U.S.-born citizens, 32.78% of foreign-born citizens, and 60.4% of non-citizens. Foreign-born adolescents also tended to have lower socioeconomic status than U.S.-born citizen adolescents. U.S.-born citizens were significantly less likely to live below the 200% of the Federal Poverty Level (40.3%) compared to both foreign-born citizens and non-citizens (43.4% and 75.4%, respectively). However, parental levels of education were highest for foreign-born U.S. citizen adolescents, with 50.4% of families having a college degree or more, compared with 38.7% of U.S.-born citizens and 27.8% of non-citizens. Unadjusted means indicate that non-citizens had significantly better mental health compared to U.S.-born citizens on all three outcome measures. Foreign-born citizens had significantly better outcomes compared to U.S.-born citizens on likely psychological problem and emotional difficulty, but not total SDQ score.

**Table 1:** Descriptive statistics by immigrant status, NHIS 2010–2016.

	U.S.-Born Citizen		Foreign-Born U.S. Citizen		Non-Citizen	
	N = 36,937		N = 1,085		N = 1,896	
	Mean or %	SE	Mean or %	SE	Mean or %	SE
**Mental Health Outcomes**						
Mental Health Severity (SDQ)	1.76	0.015	1.579	0.073	1.42*	0.043
Likely Psychological Problem	4.85	0.002	4.44	0.008	2.22*	0.004
Emotional Difficulty	22.21	0.003	19.03*	0.016	9.50*	0.009
**Age**						
10–12	38.03	0.004	27.60*	0.016	28.29*	0.015
13–15	37.41	0.003	39.73*	0.019	39.73*	0.016
16–17	24.56	0.003	32.67*	0.017	32.67*	0.015
**Race**						
Non-Hispanic, White	59.01	0.005	29.03*	0.020	11.61*	0.013
Non-Hispanic, Black	15.01	0.004	11.81*	0.013	8.31*	0.009
Non-Hispanic, Asian	3.59	0.001	21.94*	0.016	18.96*	0.013
Hispanic	20.53	0.004	32.78*	0.019	60.42*	0.004
Non-Hispanic, Other	1.87	0.002	4.44*	0.010	0.70*	0.002
**Gender**						
Male	51.20	0.004	44.44*	0.020	51.11	0.015
Female	48.80	0.004	55.56*	0.020	48.89	0.015
**Income**						
<200% FPL	40.30	0.005	43.42*	0.022	75.39*	0.015
200–399% FPL	29.95	0.004	27.36*	0.019	13.44*	0.010
400+% FPL	29.75	0.005	29.22*	0.021	11.17*	0.011
**Max Household Education**						
High school or less	26.86	0.004	21.51*	0.019	54.74*	0.018
Some College	34.75	0.004	28.08*	0.018	17.46*	0.011
College or more	38.39	0.005	50.41*	0.021	27.80*	0.016

Source: National Health Interview Survey, 2010–2016

Note: All missing data dropped was from the sample. Standard errors were calculated using Taylor Series Linearization,

* = P <0.05.

Table 2 presents the main results of the multivariable regression models (complete regression results are available in the S1 Table). On an adjusted basis, foreign-born citizens had similar mental health status as compared to U.S.-born adolescents on all three measures. However, non-citizens’ outcomes were significantly different from those of U.S.-born citizens. Non-citizens had a mental health severity score (SDQ) that was 0.35 units lower (indicating better mental health) than that of U.S.-born citizens (95% CI: 0.25–0.45), a 19.9% reduction compared to the U.S-born citizen mean. This association is larger than the association of living in a household where at least one member had a college degree or more compared to a household where the maximum education is a high school degree ((0.23 units lower, 95% CI: 0.16–0.31). Non-citizens also exhibited a lower risk of having a psychological problem (OR: 0.46, 95% CI: 0.31–0.67) and emotional difficulties (OR: 0.45, 95% CI: 0.37–0.55) compared to U.S.-born citizens.

**Table 2:** Mental health outcomes of adolescents (Age 10–17) by immigration status, NHIS 2010–2016.

	Mental Health Severity			Likely Psychological Problem			Emotional Difficulty		
Citizenship Status	Coeff.	SE	P-Value	OR	SE	P-Value	OR	SE	P-Value
**U.S.-Born Citizen**	**Reference**			Reference			Reference		
**Foreign-Born Citizen**	-0.05	0.076	0.49	1.08	0.219	0.69	1.06	0.116	0.62
**Non-Citizen**	-0.35*	0.050	0.00	0.46*	0.088	0.00	0.45*	0.046	0.00

Source: National Health Interview Survey, 2010–2016

Note: All missing data dropped from the sample, Standard errors were calculated using Taylor Series Linearization,

* = P <0.05

SE = standard error, OR = odds ratio, Coef = coefficient. Complete regression results are available in the S1 Table.

As shown in the S1 Table, coefficients for other covariates were all the size and direction we expected. For example, family income above 400% of the poverty line was associated with an SDQ score 0.60 units lower than for those with family incomes below 200%.

Table 3 shows models predicting each mental health variable separately by race/ethnicity (non-Hispanic white, non-Hispanic black, non-Hispanic Asian, Hispanic). The analysis compares the race-specific U.S. citizen category to the race specific foreign-born and non-citizen groups. This series of race-specific regressions revealed better mental health outcomes among non-citizens of each race group compared to US-born and foreign-born citizens, although statistical power was reduced due to smaller sample sizes. Results suggest that the main results were not driven by the experience of particular immigrant subgroups, but instead reflect a broader pattern of non-citizen advantage in adolescent mental health.

**Table 3:** Mental health outcomes of adolescents (10–17) by immigration status and race, NHIS 2010–2016.

	Mental Health Severity			Likely Psychological Problem			Emotional Difficulty		
	Coeff.	SE	P-Value	OR	SE	P-Value	OR	SE	P-Value
**U.S.-Born Citizen**	Reference			Reference			Reference		
**Foreign Born**									
Non-Hispanic, White	0.24	0.158	0.12	1.96*	0.578	0.02	1.33	0.277	0.17
Non-Hispanic, Black	-0.22	0.119	0.28	0.63	0.471	0.54	0.66	0.212	0.20
Non-Hispanic, Asian	-0.24	0.133	0.08	1.28	0.818	0.70	1.34	0.354	0.27
Hispanic	-0.05	0.108	0.66	0.62	0.156	0.18	0.93	0.164	0.66
**Non-Citizen**									
Non-Hispanic, White	-0.51*	0.151	0.01	0.05*	0.387	0.00	0.31*	0.278	0.00
Non-Hispanic, Black	-0.42*	0.119	0.00	0.36	0.212	0.08	0.32*	0.089	0.00
Non-Hispanic, Asian	-0.31*	0.107	0.00	0.21*	0.154	0.04	0.32*	0.101	0.00
Hispanic	-0.25*	0.067	0.00	0.70	0.156	0.11	0.58*	0.068	0.00

Source: National Health Interview Survey, 2010–2016

Note: All missing data dropped from the sample. Standard errors were calculated using Taylor Series Linearization. Estimates came from separate race-specific regressions where the reference group is the race specific U.S. citizenship category.

* = Significantly different from U.S-Born Citizens (P<0.05)

**Fig 1:**
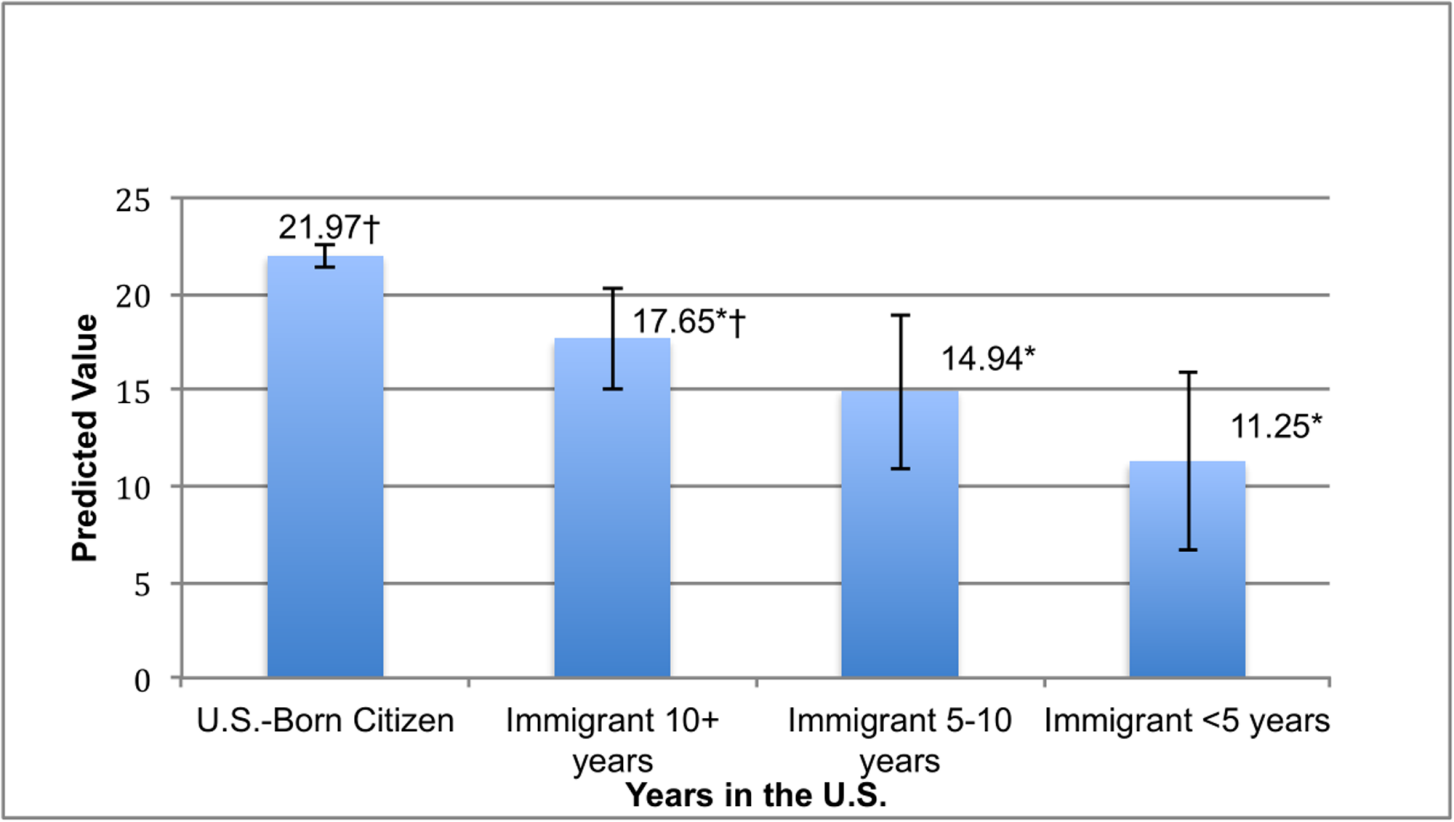
Adjusted rates of emotional difficulty by time in the U.S. Data Source: National Health Interview Survey, 2010–2016. All missing data dropped from the sample. Standard errors were calculated using Taylor Series Linearization. Estimates came from a logistic regression model. Error bars represent the 95% confidence interval, * = significantly different from U.S-Born Citizens (P<0.05), † = significantly different from immigrants living in the U.S>5 years (P<0.05).

Fig 1 reports adjusted rates of emotional difficulty for U.S.-born citizens and for immigrants whose families have been in the U.S. for varying lengths of time. The immigrant group combined foreign-born citizens and non-citizens. Foreign-born citizens and non-citizens were grouped together to increase statistical power and because the effect of duration was thought to act similarly for both groups. Each duration group had a lower adjusted rate of emotional distress compared to U.S. citizens. However, the difference between the native and foreign born diminished, in a linear fashion, as duration in the U.S. increased. Immigrants who have been here the longest (10+ years) compared to those who more recently arrived (<5 years) had a significantly higher prevalence of emotional difficulty.

In the Supplementary tables we report the results of several sensitivity tests. First, we removed Puerto Ricans from the Hispanic category given that they are citizens from birth, but may nonetheless by perceived as immigrants or non-citizens (see S5 Table). Removing them did not change our conclusions. Results were also robust to the inclusion of language of interview in the main regression and the exclusion of poverty, which may operate as a mediator (see S3 and S4 Tables). Our next sensitivity test examined if our conclusions changed when we restricted the data to the pre-ACA period (2010–2013) (see S2 Table). Given that the ACA led to large gains in insurance status, but restricted benefits to citizens and legal immigrants residing in the US longer than 5 years, results may have differed before and after the ACA. However, we obtained similar results when excluding the post-ACA years. [25]

## Discussion

Our study examined differences in mental health between U.S.-born and foreign-born adolescents by citizenship status. We found that non-citizen adolescents have a consistent advantage in self-reported mental health compared to U.S.-born citizen adolescents, despite facing substantial socioeconomic disadvantage. This finding is consistent with the broader “immigrant paradox” observed for other outcomes.[13]

The mental health advantage we observed for non-citizens did not apply to foreign-born citizens. While foreign-born citizens were significantly less likely than the U.S.-born to be classified as having a likely psychological problem or emotional difficulty on an unadjusted basis, these associations were completely explained by their observed demographic and socioeconomic position. Compared to U.S.-born citizens, foreign-born citizen adolescents are older, more likely to be female, and more likely to live in highly educated households.

While explaining the differences we observed was beyond the scope of our study, the reasons why non-citizen adolescents have better health-outcomes clearly have important public health implications. There are three broad potential explanations. First, the “true” prevalence could in fact be similar across groups and it is the reporting of mental health problems that differs. While, there is vast cultural variability across immigrant groups, it is possible that the differences in mental health outcomes we observed are due to cultural differences in recognition or reporting of mental health problems, which are also correlated with immigration status.[26] Language barriers, the level of acculturation, and stigmatization around mental health issues may be reflected in reporting mental health outcomes we observed among non-citizens.[27] However, the SDQ had been validated across diverse populations suggesting that this may not be a substantial issue.[23]

Second, the mental health advantage of non-citizen adolescents may reflect selective migration, often called the “Healthy Immigrant Effect.”[28] Immigrants do not reflect a random sample of the countries from which they came. Given the difficulty associated with moving to the United States, migrants are likely to be selected for robust psychological well-being, and are likely to represent a more motivated and resilient subset of their sending countries population.[28] There is evidence that health selectivity of new immigrants varies considerably across countries,[29] but the extent to which mental health selection might vary across sending countries is unknown. In other studies of the “immigrant paradox”, the “healthy immigrant effect” has been shown to explain the Mexican immigrant advantage in functional limitations.[28] Importantly, the presence of the healthy immigrant effect means that the mental health status of the foreign born groups we observed is unlikely to generalize to the average resident of a sending country.

Finally, better-observed mental health outcomes among non-citizen adolescents may reflect real protective factors potentially associated with social and familial support in non-citizen households and neighborhoods. Familism, the deeply ingrained sense of belonging rooted in family relationships, is a strong predictor of adolescent mental health in Latino youth and may be true for other non-citizen populations.[30] For example, research suggests that closeness with parents; religiosity and social support in immigrant families contribute to an enhanced sense well-being in this population.[8] Furthermore, the strong link between the migration process and migrant social networks underscores the fact immigrants arrive often by virtue of existing social relationships.[31] These strong relations may serve as a protective factor that helps immigrant children and adolescents cope with the stress of immigration and cultural assimilation. If such factors do indeed protect against mental health problems, there is promise in developing programs and policies that foster these characteristics in other communities and maintain them in immigrant populations.

Understanding why non-citizens benefit from a mental health advantage, but not foreign-born citizens will also be important. Table 1 suggests that non-citizens are five times less likely to be non-Hispanic White, twice as likely to live in low-income families, and three times as likely to live in low educated households. This substantial difference in observed characteristics suggests that non-citizens may also be different on unobserved characteristics. Acculturation is one clear attribute for which citizenship status is likely an important marker. Indeed, we found evidence that mental health advantage of the foreign-born as a whole deteriorates with longer time spent in the U.S.

In addition to unraveling the reasons behind the outcomes we observed, measuring the longer-run benefits of reduced risk for mental health problems among non-citizen youth is a promising area for future work. For example, better mental health among immigrant adolescents may be one reason we observe fewer health problems during adulthood in this population compared to U.S.-born citizens. Mental health may function as an important mediator leading to lower rates of smoking among immigrant adults, which has been identified as a primary explanation for their mortality advantage over U.S.-born adults.[32] In addition, research has suggested that greater rates of mental health problems among U.S.-born Mexican Americans compared with Mexican immigrants may reflect a higher risk of substance abuse disorders in the U.S.-born population.[33]

### Limitations

Our study had limitations. First, the cross-sectional design prohibited tracking mental health outcomes of individuals over time. As a result, we were unable to determine if mental health declined with duration of residence at the individual level. Of note, the use of the Additionally, our mental health outcomes came from proxy reports of a knowledgeable adult in the household and thus might be reflective of the proxy’s social distance to the child, their own mental health status, or their attitudes about mental health problems.[24] Finally, immigrants who are undocumented or fearing their legal status may be reluctant to respond to surveys such as the NHIS in the first place, leaving a portion of the immigrant population unobserved in this analysis.

## Public health implications

While our results suggested that non-citizen adolescents fared better than their U.S.-born peers, nearly 1 in 10 non-citizen youth and 1 in 5 foreign-born citizens experienced an emotional difficulty. Some of the mental health challenges that immigrant youth face could indeed stem from the current immigration policy environment. For example, a recent study of the Deferred Action for Childhood Arrivals (DACA) program improved the mental health status of those eligible.[34] Another study found that mothers’ eligibility for DACA protection led to a significant improvement in their children’s mental health suggesting important intergenerational effects.[35]

Furthermore, it is well documented that mental health service utilization in immigrant populations is far lower than it is for U.S.-born citizens.[27] Lee and Matejkowski [36] found that both non-citizens and foreign-born citizens were 40% less likely to use any mental health service than U.S.-born citizens. Given that foreign-born adolescent citizens have similar mental health outcomes to their U.S.-born counterparts, after accounting for their socio-demographic characteristics, this could have negative implications for the foreign-born population. Other factors such as English language proficiency, cultural attitudes toward mental health, and family support also impact immigrant’s likelihood of seeking mental health services. [37] Immigrants may also be less likely to seek help if they view challenges and hardships as a normal part of life.[27] Thus, policy action is needed first to reduce financial barriers to care that are particularly pronounced for non-citizens that often are not eligible for public support. These barriers directly affect non-citizens and indirectly affect their citizen family members. In addition to financial support, culturally appropriate education and care is needed to serve immigrant communities in a patient centered manner. Further analysis of immigrant subgroups would provide a better understanding of the mental health outcomes in the non-citizen population. Future monitoring of these results is warranted.

## Supporting information

S1 Table Mental health outcomes of adolescents (10–17) by immigration category, including covariates, NHIS 2010–2016. S1 Table shows the same regression as Table 2 in the text including the covariates for comparison. The covariate results are the size and direction we expected and are almost all significant.
(DOCX)

S2 Table Mental health outcomes of adolescents (10–17) by immigration category, NHIS 2010–2013. S2 Table shows the same regression as Table 2 in the text with the years 2014–2016 removed from the sample to account for the implementation of the Affordable Care Act in the regression results. The results hold true for all three variables.
(DOCX)

S3 Table Mental health outcomes of adolescents (10–17) by immigration category, excluding poverty, NHIS 2010–2016. S3 Table shows the same regression as Table 2 in the text with poverty excluded from the regression model. The results hold true for all three variables.
(DOCX)

S4 Table Mental health outcomes of adolescents (10–17) including language in the regression, NHIS 2010–2016. S4 Table shows the same regression as Table 2 in the text with the language of the interview added to the regression. The results remain significant for all three mental health variables.
(DOCX)

S5 Table Mental health outcomes of adolescents (10–17) based on SDQ scale with Puerto Ricans separated from Hispanic ethnicity in the regression, NHIS 2010–2016. S5 Table shows the same regression from Table 2 in the text with Puerto Rican as a separate ethnicity category from the rest of the Hispanic population. Though Puerto Ricans are U.S. citizens by birth, we wanted to see if there was a difference between those born on the U.S. mainland and those born in the islands. The results remained significant for the mental health variables with this change.
(DOCX)

## References

[1] Hollenstein T, Lougheed JP. Beyond storm and stress: Typicality, transactions, timing, and temperament to account for adolescent change. Am Psychol. 2013;68: 444–454. doi: 10.1037/a0033586 23915399 10.1037/a0033586

[2] Behere AP, Basnet P, Campbell P. Effects of Family Structure on Mental Health of Children: A Preliminary Study. Indian J Psychol Med. 2017;39: 457–463. doi: 10.4103/0253-7176.211767 28852240 10.4103/0253-7176.211767 PMC5559994

[3] Kessler RC, Berglund P, Demler O, Jin R, Merikangas KR, Walters EE. Lifetime Prevalence and Age-of-Onset Distributions of DSM-IV Disorders in the National Comorbidity Survey Replication. Arch Gen Psychiatry. 2005;62: 593–602. doi: 10.1001/archpsyc.62.6.593 15939837 10.1001/archpsyc.62.6.593

[4] Patel V, Flisher AJ, Hetrick S, McGorry P. Mental health of young people: a global public-health challenge. The Lancet. 2007;369: 1302–1313. doi: 10.1016/S0140-6736(07)60368-7 10.1016/S0140-6736(07)60368-7 17434406

[5] Lopez MH, Passell J, Rohal M. Modern Immigration Wave Brings 59 Million to U.S., Driving Population Growth and Change Through 2065: Views of Immigration’s Impact on U.S. Society Mixed [Internet]. Pew Research Center; 2015. http://assets.pewresearch.org/wp-content/uploads/sites/7/2015/09/2015-09-28_modern-immigration-wave_REPORT.pdf

[6] Singh GK, Yu SM, Kogan MD. Health, chronic conditions, and behavioral risk disparities among U.S. immigrant children and adolescents. Public Health Rep Wash DC 1974. 2013;128: 463–479. doi: 10.1177/003335491312800606 24179258 10.1177/003335491312800606 PMC3804090

[7] Turner RJ, Gil AG. Psychiatric and substance use disorders in South Florida: racial/ethnic and gender contrasts in a young adult cohort. Arch Gen Psychiatry. 2002;59: 43–50. 11779281 10.1001/archpsyc.59.1.43

[8] Harker K. Immigrant Generation, Assimilation, and Adolescent Psychological Well-Being. Soc Forces. 2001;79: 969–1004.

[9] Yoshikawa H, Suarez-Orozco C, Gonzales R. Unauthorized Status and Youth Development in the United States. Consensus Statement of the Society for Research on Adolescence. 2016;27: 4–16. 10.1111/jora.12272 28498536

[10] Siemons R, Raymond-Flesh M, Auerswald CL, Brindis CD. Coming of Age on the Margins: Mental Health and Wellbeing Among Latino Immigrant Young Adults Eligible for Deferred Action for Childhood Arrivals (DACA). J Immigr Minor Health. 2017;19: 543–551. doi: 10.1007/s10903-016-0354-x 26852235 10.1007/s10903-016-0354-x

[11] Cornelius WA. Death at the Border: Efficacy and Unintended Consequences of US Immigration Control Policy. Popul Dev Rev. 2001;27: 661–685. doi: 10.1111/j.1728-4457.2001.00661.x

[12] Jasso G, Douglas S. M. Immigrant health: Selectivity and acculturation [Internet]. IFS Working Papers, Institute for Fiscal Studies (IFS); 2004. https://www.econstor.eu/handle/10419/71469

[13] Blue L, Fenelon A. Explaining low mortality among US immigrants relative to native-born Americans: the role of smoking. Int J Epidemiol. 2011;40: 786–793. doi: 10.1093/ije/dyr011 21324939 10.1093/ije/dyr011 PMC3147070

[14] Moscicki EK, Locke BZ, Rae, Boyd. Depressive Symptoms Among Mexican Americans: The Hispanic Health and Nutrition Examination Survey. Am J Epidemiol. 1989;130: 348–360. doi: 10.1093/oxfordjournals.aje.a115341 2750730 10.1093/oxfordjournals.aje.a115341

[15] Acevedo-Garcia D, Bates LM, Osypuk TL, McArdle N. The effect of immigrant generation and duration on self-rated health among US adults 2003–2007. Soc Sci Med 1982. 2010;71: 1161–1172. doi: 10.1016/j.socscimed.2010.05.034 20624666 10.1016/j.socscimed.2010.05.034

[16] Pottie K, Dahal G, Georgiades K, Premji K, Hassan G. Do First Generation Immigrant Adolescents Face Higher Rates of Bullying, Violence and Suicidal Behaviours Than Do Third Generation and Native Born? J Immigr Minor Health. 2015;17: 1557–1566. doi: 10.1007/s10903-014-0108-6 25248622 10.1007/s10903-014-0108-6 PMC4562994

[17] Sirin SR, Ryce P, Gupta T, Rogers-Sirin L. The role of acculturative stress on mental health symptoms for immigrant adolescents: A longitudinal investigation. Dev Psychol. 2013;49: 736–748. doi: 10.1037/a0028398 22563676 10.1037/a0028398

[18] Shear MD, Davis JH. Trump Moves to End DACA and Calls on Congress to Act. The New York Times. 5 Sep 2017. https://www.nytimes.com/2017/09/05/us/politics/trump-daca-dreamers-immigration.html

[19] Gee GC, Ryan A, Laflamme DJ, Holt J. Self-Reported Discrimination and Mental Health Status Among African Descendants, Mexican Americans, and Other Latinos in the New Hampshire REACH 2010 Initiative: The Added Dimension of Immigration. Am J Public Health. 2006;96: 1821–1828. doi: 10.2105/AJPH.2005.080085 17008579 10.2105/AJPH.2005.080085 PMC1586129

[20] Blewett L, Rivera J, King, William. IPUMS Health Surveys: National Health Interview Survey, Version 6.2 [Internet]. University of Minnesota; 2016. https://ihis.ipums.org/ihis/people.shtml

[21] Rajmil L, Fernández E, Gispert R, Rué M, Glutting JP, Plasència A, et al Influence of proxy respondents in children’s health interview surveys. J Epidemiol Community Health. 1999;53: 38–42. doi: 10.1136/jech.53.1.38 10326051 10.1136/jech.53.1.38 PMC1756765

[22] Van Roy B, Veenstra M, Clench-Aas J. Construct validity of the five-factor Strengths and Difficulties Questionnaire (SDQ) in pre-, early, and late adolescence. J Child Psychol Psychiatry. 2008;49: 1304–1312. doi: 10.1111/j.1469-7610.2008.01942.x 19120709 10.1111/j.1469-7610.2008.01942.x

[23] Achenbach TM, Becker A, Döpfner M, Heiervang E, Roessner V, Steinhausen H-C, et al Multicultural assessment of child and adolescent psychopathology with ASEBA and SDQ instruments: research findings, applications, and future directions. J Child Psychol Psychiatry. 2008;49: 251–275. doi: 10.1111/j.1469-7610.2007.01867.x 18333930 10.1111/j.1469-7610.2007.01867.x

[24] Bourdon KH, Goodman R, Rae DS, Simpson G, Koretz DS. The Strengths and Difficulties Questionnaire: U.S. Normative Data and Psychometric Properties. J Am Acad Child Adolesc Psychiatry. 2005;44: 557–564. doi: 10.1097/01.chi.0000159157.57075.c8 15908838 10.1097/01.chi.0000159157.57075.c8

[25] Stimpson JP, Wilson FA, Su D. Unauthorized Immigrants Spend Less Than Other Immigrants And US Natives On Health Care. Health Aff (Millwood). 2013;32: 1313–1318. doi: 10.1377/hlthaff.2013.0113 23759790 10.1377/hlthaff.2013.0113

[26] Jimenez DE, Bartels SJ, Cardenas V, Daliwal SS, Alegría M. Cultural Beliefs and Mental Health Treatment Preferences of Ethnically Diverse Older Adult Consumers in Primary Care. Am J Geriatr Psychiatry. 2012;20: 533–542. doi: 10.1097/JGP.0b013e318227f876 21992942 10.1097/JGP.0b013e318227f876 PMC3258470

[27] Kung WW. Chinese Americans’ Help Seeking for Emotional Distress. Soc Serv Rev. 2003;77: 110–134. doi: 10.1086/345707

[28] Bostean G. Does Selective Migration Explain the Hispanic Paradox?: A Comparative Analysis of Mexicans in the U.S. and Mexico. Sociol Fac Artic Res. 2013; doi: 10.1007/s10903-012-9646-y 22618355 10.1007/s10903-012-9646-y PMC3901783

[29] Akresh IR, Frank R. Health Selection Among New Immigrants. Am J Public Health. 2008;98: 2058–2064. doi: 10.2105/AJPH.2006.100974 18309141 10.2105/AJPH.2006.100974 PMC2636435

[30] Smokowski PR, Chapman MV, Bacallao ML. Acculturation Risk and Protective Factors and Mental Health Symptoms in Immigrant Latino Adolescents. J Hum Behav Soc Environ. 2007;16: 33–55. doi: 10.1300/10911350802107710

[31] Riosmena F, Massey DS. Pathways to El Norte: origins, destinations, and characteristics of Mexican migrants to the United States. Int Migr Rev. 2012;46: 3–36. 22666876 10.1111/j.1747-7379.2012.00879.x PMC3816114

[32] Fenelon A. Revisiting the Hispanic mortality advantage in the United States: the role of smoking. Soc Sci Med 1982. 2013;82: 1–9. doi: 10.1016/j.socscimed.2012.12.028 23453311 10.1016/j.socscimed.2012.12.028 PMC3588600

[33] Escobar JI, Hoyos Nervi C, Gara MA. Immigration and mental health: Mexican Americans in the United States. Harv Rev Psychiatry. 2000;8: 64–72. 10902095

[34] Venkataramani AS, Shah SJ, O’Brien R, Kawachi I, Tsai AC. Health consequences of the US Deferred Action for Childhood Arrivals (DACA) immigration programme: a quasi-experimental study. Lancet Public Health. 2017; doi: 10.1016/S2468-2667(17)30047-6 10.1016/S2468-2667(17)30047-6 PMC6378686 29253449

[35] Hainmueller J, Lawrence D, Martén L, Black B, Figueroa, Hotard, et al Protecting unauthorized immigrant mothers improves their children’s mental health. Science. 2017; 5893. doi: 10.1126/science.aan5893 28860206 10.1126/science.aan5893 PMC5990252

[36] Lee S, Matejkowski J. Mental Health Service Utilization Among Noncitizens in the United States: Findings From the National Latino and Asian American Study. Adm Policy Ment Health Ment Health Serv Res. 2012;39: 406–418. doi: 10.1007/s10488-011-0366-8 21755392 10.1007/s10488-011-0366-8

[37] Derr AS. Mental Health Service Use Among Immigrants in the United States: A Systematic Review. Psychiatr Serv. 2016;67: 265–274. doi: 10.1176/appi.ps.201500004 26695493 10.1176/appi.ps.201500004 PMC5122453

