# Supplementary material for: Immigration, citizenship, and the mental health of adolescents
Source: PLoS One. 2018 May 3;13(5):e0196859. doi: 10.1371/journal.pone.0196859 (PMC5933703; doi:10.1371/journal.pone.0196859)
Supplement: S1 Table — shows the same regression as Table 2 in the text including the covariates for comparison. The covariate results are the size and direction we expected and are almost all significant. (DOCX) [file pone.0196859.s001.docx]

**S1 Table: Mental Health Outcomes of Adolescents (10-17) by Immigration Category, Including Covariates, NHIS 2010-2016**. S1 Table shows the same regression as Table 2 in the text including the covariates for comparison. The covariate results are the size and direction we expected and are almost all significant.
