# Supplementary material for: Immigration, citizenship, and the mental health of adolescents
Source: PLoS One. 2018 May 3;13(5):e0196859. doi: 10.1371/journal.pone.0196859 (PMC5933703; doi:10.1371/journal.pone.0196859)
Supplement: S2 Table — shows the same regression as Table 2 in the text with the years 2014–2016 removed from the sample to account for the implementation of the Affordable Care Act in the regression results. The results hold true for all three variables. (DOCX) [file pone.0196859.s002.docx]

**S2 Table: Mental Health Outcomes of Adolescents (10-17) by Immigration Category, NHIS 2010-2013.** S2 Table shows the same regression as Table 2 in the text with the years 2014-2016 removed from the sample to account for the implementation of the Affordable Care Act in the regression results. The results hold true for all three variables.
