# Supplementary material for: Immigration, citizenship, and the mental health of adolescents
Source: PLoS One. 2018 May 3;13(5):e0196859. doi: 10.1371/journal.pone.0196859 (PMC5933703; doi:10.1371/journal.pone.0196859)
Supplement: S4 Table — shows the same regression as Table 2 in the text with the language of the interview added to the regression. The results remain significant for all three mental health variables. (DOCX) [file pone.0196859.s004.docx]

**S4 Table: Mental Health Outcomes of Adolescents (10-17) Including Language in the Regression, NHIS 2010-2016.** S4 Table shows the same regression as Table 2 in the text with the language of the interview added to the regression. The results remain significant for all three mental health variables.
