# Supplementary material for: Immigration, citizenship, and the mental health of adolescents
Source: PLoS One. 2018 May 3;13(5):e0196859. doi: 10.1371/journal.pone.0196859 (PMC5933703; doi:10.1371/journal.pone.0196859)
Supplement: S5 Table — shows the same regression from Table 2 in the text with Puerto Rican as a separate ethnicity category from the rest of the Hispanic population. Though Puerto Ricans are U.S. citizens by birth, we wanted to see if there was a difference between those born on the U.S. mainland and those born in the islands. The results remained significant for the mental health variables with this change. (DOCX) [file pone.0196859.s005.docx]

**S5 Table: Mental Health Outcomes of Adolescents (10-17) Based on SDQ scale with Puerto Ricans Separated from Hispanic Ethnicity in the Regression, NHIS 2010-2016.** S5 Table shows the same regression from Table 2 in the text with Puerto Rican as a separate ethnicity category from the rest of the Hispanic population. Though Puerto Ricans are U.S. citizens by birth, we wanted to see if there was a difference between those born on the U.S. mainland and those born in the islands.  The results remained significant for the mental health variables with this change.
